# Maternal behavior in basic science: translational research and clinical applicability

**DOI:** 10.1590/S1679-45082013000200021

**Published:** 2013

**Authors:** Gabriel Natan Pires, Sergio Tufik, Márcia Giovenardi, Monica Levy Andersen

**Affiliations:** 1 Universidade Federal de São Paulo, São Paulo, SP, Brazil; 2 Universidade Federal de Ciências da Saúde de Porto Alegre, Porto Alegre, RS, Brazil

**Keywords:** Maternal behavior, Translational medical research, Motherchild relations, Paternal behavior, Models, animal

## Abstract

Clinical aspects of the mother-infant relationship and related issues are well studied and very relevant to medical practice. Nevertheless, some approaches in this context cannot plausibly be investigated in humans due to their ethical implications and to the potential harm to the mother's and child's health. Studies on maternal behavior in animals have evident importance to some clinical fields, such as psychiatry and psychology, particularly considering topics, including mother-infant relationship, postpartum depression, cognitive and behavioral development of children, and associated issues. Hence, this theoretical article draws attention to the clinical applicability of studies about maternal behavior in animals to psychobiology, taking into account a translational perspective.

## RESUMO

Os aspectos clínicos da relação materno-infantil e assuntos a eles associados são suficientemente bem estudados e conhecidos, e de grande importância à prática médica. Contudo, algumas abordagens de pesquisa, nesse contexto, não podem ser feitas em seres humanos de forma plausível, devido a implicações éticas e ao potencial dano à saúde, tanto da mãe quanto da criança. Os estudos sobre comportamento materno em animais apresentam importância evidente em áreas clínicas, como psiquiatria e psicologia, particularmente quando considerados temas como relação materno-infantil, depressão pós-parto, desenvolvimento cognitivo e comportamental infantil, entre outros. Portanto, este ensaio teórico chama atenção para a aplicabilidade clínica de estudos sobre comportamento materno feitos em animais de experimentação em psicobiologia, considerando uma perspectiva translacional.

## INTRODUCTION

Parturition represents a dramatic transitional moment for the mother-infant relationship. From that point forward, the intimate anatomical and physiological connections between mother and child experienced during gestation are replaced by lactation and various other behavioral and physiological interactions. These interactions take place to maintain mother and child as a development dyad throughout breastfeeding, weaning and beyond^(1)^. The transitions entailed by parturition are of interest to several clinical areas, such as obstetrics, pediatrics, and endocrinology. Furthermore, behavioral aspects of the mother-infant relationship and the ways in which it can be altered due to events during pregnancy are of special interest to psychiatry. From the viewpoint of this clinical field, the breastfeeding period and the maternal bond represent a broad topic of interest. Maternal behavior, postpartum and perinatal depression, maternal fatigue, and the mother-infant relationship *per se* are research topics that stand out^(1–3)^.

Some clinical aspects of mother-infant relationship and related issues are well-documented. However, some approaches to research in this area cannot be employed in humans due to their ethical implications and to potential harm they pose for mother and child health. Thus, basic research is an effective and useful way to dissect this relationship. In the following sections, we discuss the reasons why the use of animal models to assess mother-infant relationship in humans is valuable, describe the most frequently used models and the main findings of studies that use them, and present the limitations of these techniques.

### Why use animal models to assess the mother-infant relationship in humans?

The logical sequence of pregnancy, parturition, lactation, and weaning in humans is interspersed by several physiological and hormonal changes; some of them are abrupt, like parturition, and others gradual, like weaning. Furthermore, psychological changes occur constantly along this continuum, and these alterations are deeply involved in, e.g., the onset of postpartum blues and depression, development of the mother-infant unit, and child cognitive development^(4)^.

Although several issues related to these physiological and psychological alterations can be studied in humans, many others are better demonstrated in animals, especially in rodents (rats and mice), through maternal behavior models. Indeed, the investigation of maternal behavior in animals has proven valuable to understand the mother-infant relationship and some associated themes. Maternal behavior in rodents is well-described in the literature. In brief, it is composed of four major features: retrieval behavior, nest building, nursing behavior, and pup grooming^(5)^. Because these behavioral patterns are well characterized, rodent maternal behavior can be quantified in response to various contexts or treatments. Moreover, animal models have inherent advantages for generic research, including their low cost, the strict control of variables, and the short life cycle.

The study of maternal behavior in animals allows manipulating variables that cannot possibly be investigated in humans. Among these factors are administration of drugs (e.g.: haloperidol, clozapine^(6)^, amisulpride, and aripiprazole^(7)^), administration or manipulation of hormones or neurotransmitters (e.g.: oxytocin^(8)^, and progesterone^(9)^), and use of techniques that may be stressful both to the lactating female and to the litter (e.g.: maternal separation^(10)^, neonatal handling^(11)^, chronic pain^(12)^, and maternal hypoxia^(13)^). Collectively, the inherent characteristics of animal research, the well-described patterns of maternal behavior, and the possibility of employing approaches that are not possible in humans, make maternal behavior in rodents a reliable and efficient model of human maternal behavior from the perspective of translational science. Additionally, these characteristics offer the opportunity to provide new insights into the mother-infant relationship in humans. In fact, several findings in the context of rat maternal behavior have implications for clinical sciences, as discussed in the following sections.

### Animal models of maternal behavior

Efficient models of maternal behavior have been developed for use in basic research. These models, in general, are based on the four aforementioned major features of this behavior and on patterns of initiation and conclusion of maternal care. Among these protocols are those employed by Lonstein and Stern^(14)^, which is based on latency, frequency, and duration of several behavioral patterns; by Felicio et al.^(15)^, based on latency of specific behaviors and the presence of full maternal behavior; and by Myers et al.^(16)^, which allows classifying maternal behavior considering frequency of grooming and licking. Recently, Carola et al.^(17)^ presented a model that differs from those cited above, employing a mathematical analysis distinct from the traditional statistical approaches for behavioral studies. This model, named “Hidden Markov model analysis of maternal behavior patterns” describes, in detail, the ordering, clustering and transitions among several behavioral patterns, thus promoting a complete evaluation of maternal behavior as a whole. However, despite promising, this model needs further examination and replication to become a useful experimental tool.

Furthermore, maternal aggression, another aspect of maternal behavior in animals, can be investigated by specific protocols derived from the resident-intruder paradigm^(11)^. Finally, Lucion and Almeida^(18)^ proposed a mixed model that enables analysing both maternal behavior and maternal aggression in the same protocol.

### Basic research and major findings related to clinical applicability

By means of these protocols, several studies have been performed, yielding many interesting results with clinical applicability from the perspective of translational science.

Probably the most clinically relevant finding from basic research on maternal behavior concerns the importance of the environmental milieu to the behavioral phenotype. The maternal behavioral phenotype is defined as heritable not purely through genetic pathways, but by a nongenomic behavioral mode of inheritance based on epigenetic and environmental factors^(19)^. This argument has been strengthened by studies reporting that, in rats, the litter acquires the same maternal behavioral patterns exhibited by the dam. After clarifying the environmental relevance to maternal behavior, it was observed that the litters of higher licking dams (with more prominent maternal behavior) when fostered with lower licking dams, grew up to be lower licking dams too, exhibiting low maternal care^(20)^. Moreover, the maternal behavior phenotype is also related to other issues, such as anxiety and cognition. In general, the litter of higher licking dams tends to be less anxious in adulthood and more efficient in learning, memory, and object recognition tasks^(21)^. Extrapolating these findings to a human context, one might hypothesize the relevance of the environment, and particularly the characteristics of the mother-infant relationship to child behavior later in life. Thus, ultimately, it can be concluded that the behavioral phenotype of an adult individual is a function of the maternal care he received from his mother during early childhood.

Such data are particularly interesting considering that some characteristics of mother-infant interactions can be passed down from human mothers to their children^(22)^. Indeed, Ogren and Lambroso^(21)^ stated that the employment of animal models of maternal behavior has become remarkably relevant to investigate the consequences of early life experiences in mental health during adulthood. Moreover, Miller stated that these findings raised in animal basic research, if extended to humans, would play determining roles in explaining relevant issues, such as the long-lasting health problems in socioeconomically disadvantaged people, drug abuse cessation, and tendency of abused children to grow up and be abusive parents like their own parents^(23)^.

Several others aspects of the relationship between maternal care and child cognitive and behavioral development have been assessed by animal experiments. For instance, studies in rodents and nonhuman primates demonstrated long-term deleterious consequences of maternal care deprivation and maternal separation for offspring's cognition and behavior. These consequences included reduced pain sensitivity, increased fearfulness, excessive aggressive patterns, impaired cognitive development, altered sensitivity and drug intake pattern^(10,24–26)^. These studies also increased our understanding about the possible mechanisms and etiology of postpartum depression in humans. Recently, Pawluski et al.^(27)^ observed a clear relation between pup contact and reduction in depressive-like behaviors in the mother. This result corroborates the hypothesis that breastfeeding is the most important contact between the human mother and child and that an early interruption of breastfeeding is correlated with an increased risk of postpartum depression^(28)^. In this sense, Smith et al.^(29)^ observed the induction of postpartum depressive-like behavior in rats submitted to stress during pregnancy, while White et al.^(2)^ reported impairment of the mother-infant relationship due to maternal fatigue and stress during pregnancy.

Several aspects of the hypotheses regarding neurobiology of maternal behavior are rooted in animal experimentation. Such is the case of the approachavoidance model of maternal behavior proposed by Rosenblatt and Mayer^(30)^, a complex model focused on the tendency of a mother to approach or avoid her offspring. This model has a strong neuroanatomical basis that was developed through basic experimentation^(1)^. Indeed, most current knowledge regarding neurobiology, neurochemistry, and neural circuitry underlying maternal behavior resulted from animal studies. This phenomenon is understandable given the difficulty of performing such assessments in humans.

**Figure 1:**
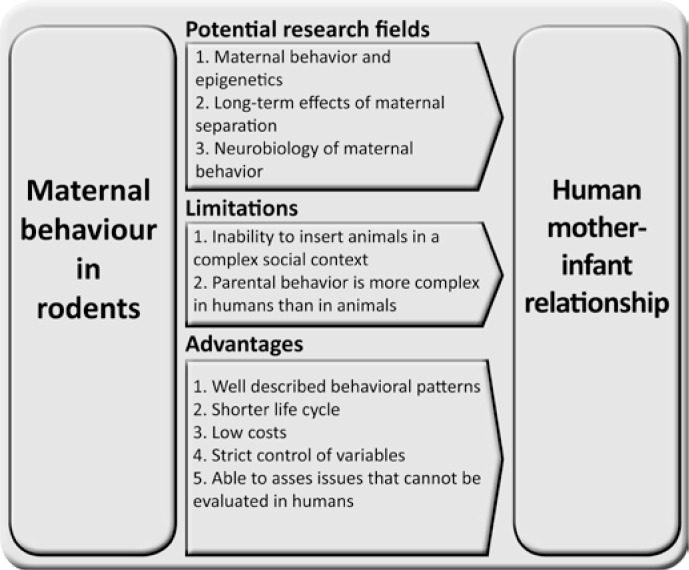
Key concepts regarding the translation of basic findings on maternal behavior to human mother-infant relationship and associated issues

Despite the evident relevance of animal experimentation as a model of mother-infant relationship in humans from a translational perspective, some concerns must be raised to ensure appropriate interpretation of these studies. Certainly, the maternal bond and parental behavior as a whole are far more complex in humans than in animals. Despite the current importance of translational research, in which animal experimentation is performed with a view toward clinical applicability, caution should be exercised when there is a psychiatric or psychological background. Indeed, it should be assumed that animals do not share the neural, cognitive, and behavioral complexity that humans possess, and lack higher cognitive functions and affective states that lead to specific psychiatric and psychological conditions in human beings. Moreover, these animals cannot be inserted into a social context that influences, for example, the maternal bond or the onset of postpartum depression. Nevertheless, the importance of basic research for knowledge on mother-infant relationship in humans is undeniable. Additionally, this relevance can be extrapolated to other areas of behavioral research. Thus, animal research as a whole, and specifically to mother-infant relationship and associated issues, is still a great investigation tool to understand the mechanisms underlying this behavior and its consequences. Furthermore, some advantages of animal research, such as low cost, shorter life cycle of animals, well-described behavioral patterns, and the ability to assess circumstances that cannot plausibly be manipulated in humans make these studies more relevant. An overview of the most significant limitations and advantages of the use of animals for translational purposes related to mother-infant relationship, in addition to the most important research fields in this context are shown in figure 1.

## CONCLUSIONS

Investigation of maternal behavior in animals has evident importance in the context of translational science to clinical areas, including psychiatry and psychology. Moreover, animal models of maternal behavior are of particular relevance when investigating some topics, such as mother-infant relationship, postpartum depression, and associated issues. Lastly, this article draws attention to the significance of maternal behavior in animals for future findings regarding the subjects discussed.
